# The Valsalva Maneuver Is Not as Effective as Lidocaine for the Attenuation of Pain on Injection of Propofol: A Randomized Controlled Study

**DOI:** 10.7759/cureus.25678

**Published:** 2022-06-05

**Authors:** Farheen Ahmed, Prakash K Dubey, Akhileshwar .

**Affiliations:** 1 Anaesthesiology, Indira Gandhi Institute of Medical Sciences, Patna, IND; 2 Emergency Medicine, Indira Gandhi Institute of Medical Sciences, Patna, IND

**Keywords:** distraction, lidocaine, valsalva maneuver, pain, complication, propofol

## Abstract

Background and objective

Lidocaine pretreatment is considered the gold standard for attenuating pain on injection of propofol. Valsalva maneuver (VM) causes baroreceptor reflex arc-induced antinociception by increasing the intrathoracic pressure. We aimed to evaluate the efficacy of VM in alleviating the pain on injection of propofol in this randomized comparative study.

Methods

A total of 90 patients were recruited for this randomized study. They were classified into two groups. Patients in group D received 5-mL 4% lidocaine in saline intravenously while the venous drainage was occluded. Then they were asked to press a rubber ball as hard as they could. Patients in group V received 5-ml saline pretreatment. They were then asked to perform VM by blowing into rubber tubing connected to a manometer and raising and holding the pressure up to 40 mmHg. The verbal response and behavioral signs were recorded with a score corresponding to no, mild, moderate, or severe pain. A t-test was performed to compare the mean of variables between the two groups. The Kolmogorov-Smirnov test was used for testing the equality of the distribution function of pain scores between the groups. Repeated measures analysis of variance (ANOVA) was performed to test the heart rate and mean arterial pressure (MAP) at different points of observation.

Results

The incidence of pain and pain scores were significantly higher among the patients in the VM group as compared to those in the lidocaine with distraction group.

Conclusions

VM performed immediately before the injection failed to attenuate the pain produced by propofol as compared to lidocaine pretreatment along with distraction.

## Introduction

Propofol is the anesthetic induction agent of choice worldwide due to its superior induction and recovery profile. A recent review suggests that three out of five patients experience pain on injection of propofol, with one of these patients reporting severe or excruciating pain [1]. Various pharmacological and non-pharmacological interventions have been introduced to alleviate this pain. Pretreatment with lidocaine is considered the gold standard among these strategies [2].

The Valsalva maneuver (VM) causes contraction of the thoracic cage, compressing the lungs. The resulting increase in intrathoracic pressure causes baroreceptor activation by the compression of the vessels within the chest [3]. It is known that the cardiopulmonary baroreceptor reflex arc or the sinoaortic baroreceptor reflex arc activation can induce antinociception [4,5]. Another option for reducing pain is by diverting attention to a non-noxious stimulus in the surrounding environment, i.e., distraction [6].

In this study, we planned to evaluate the efficacy of VM in alleviating the pain on injection of propofol in healthy adult patients by comparing it with the standard strategy, i.e., vein pretreatment with lidocaine. To isolate the possible element of distraction from VM, subjects in the lidocaine group were asked to press a ball during propofol injection.

VM has been found to be effective in alleviating venous cannulation and spinal needle insertion pain [7-10]. As VM induces bradycardia and hypotension, which may be significant following propofol injection, we also compared the hemodynamic responses before and after the intervention between the groups.

Propofol injection pain scoring described by McCririck and Hunter was used in the study to assess the pain [11]. This study compared an established pharmacological intervention for the alleviation of propofol injection pain with a non-pharmacological strategy.

## Materials and methods

This study was conducted after obtaining approval from the Institutional Ethics Committee, Indira Gandhi Institute of Medical Sciences, Patna, India (Ref: 489/IEC/2018/IGIMS), and registering the trial with the Clinical Trials Registry - India (www.ctri.nic.in) vide registration number CTRI/2019/02/017466. Valid informed consent was obtained from all participants before enrolling them in the study. This was a parallel-group, randomized clinical trial conducted between March 2019 and August 2020 at a tertiary care university hospital. The CONSORT (CONsolidated Standards of Reporting Trials) guidelines were followed in conducting this study.

The eligibility criteria for participants were as follows: patients with the American Society of Anesthesiologists (ASA) physical status 1 and 2, aged between 18 and 60 years of either sex, having the ability to comprehend and willing to hold the sphygmomanometer up to 40 mmHg for a period of at least 15 seconds [12], and those in whom propofol was indicated for induction of anesthesia.

Patients who refused to participate, those with known sensitivity to lidocaine or propofol, those on concomitant analgesic or sedative medication, and patients with an infection on the dorsum of the left hand were excluded from the study. Patients in whom VM was contraindicated (e.g., raised intracranial or intraocular pressure, glaucoma, retinopathy, middle ear pathology) and those who were unable to hold the sphygmomanometer up to 40 mmHg for a period of at least 15 seconds were also excluded.

Based on the literature review, the incidence of pain on propofol injection was reported to be around 70%, and it was assumed that a 40% reduction by the desired intervention was considered clinically as well as statistically significant [13]. At a 5% level of significance, 90% power to detect the desired difference, and 30% absolute margin of error, the sample size estimated was 45 subjects in each arm.

After their enrolment in the study, all patients were instructed about how to blow into sphygmomanometer tubing. All patients received 0.25-mg alprazolam and 150-mg ranitidine orally the previous evening. No premedication was administered on the day of surgery. The placement of standard ASA monitors was followed by the placement of a 20-gauge (20G) cannula into the largest vein on the dorsum of the left hand.

Block randomization method using a number-coded, opaque, sealed envelope was used to allocate recruited subjects into two groups. Patients in group D received 5 mL of pretreatment solution: 40-mg lidocaine in saline intravenously for a period of 10 seconds while the venous drainage was occluded with a pneumatic tourniquet (70 mmHg) on the upper arm. The occlusion was released after two minutes. Then the patients were given a rubber ball to be held in the palm of their right hand and were asked to press it as hard as they could. Patients in group V received 5-ml saline pretreatment intravenously over a period of 10 seconds while the venous drainage remained occluded for two minutes. They were then asked to perform VM by blowing into rubber tubing connected to a manometer and raising and holding the pressure up to 40 mmHg. All patients were oxygenated for three minutes before starting these maneuvers.

Fifteen seconds after starting these maneuvers, one-fourth of the total calculated dose of propofol (2.0 mg/kg body weight) kept at room temperature was administered for a period of five seconds. Propofol injection was not preceded by any analgesic or sedative. Another independent clinician assessed the level of pain on injection of propofol by asking the participants if the injection was comfortable. The verbal response and behavioral signs, such as facial grimacing, arms withdrawal, or tears, were documented. A score of 0-3, corresponding to no, mild, moderate, or severe pain, respectively, was noted [11]. Mean arterial pressure (MAP) and heart rate five minutes before the interventions, and before and after the administration of propofol were recorded. These recordings represented the baseline value, effect of Valsalva or distraction, and the combined effect of propofol and these maneuvers, respectively. The rescue medications were atropine for bradycardia (less than 50 BPM) and mephentermine for hypotension (MAP less than 20% of the baseline value). Anesthetic induction and maintenance were performed in a similar fashion thereafter in all cases.

The primary outcome of this study was to assess and compare the incidence and severity of pain on propofol injection between patients receiving lidocaine pretreatment along with distraction and those performing VM. The secondary outcome was the hemodynamic effects of these maneuvers.

Statistical analyses

All statistical analyses were performed using the statistical software Stata version 12 (StataCorp, College Station, TX). Categorical variables were expressed as frequency (%) and the chi-square test was applied to test the significance of the association between groups and variables. Continuous variables were expressed as means with 95% confidence intervals (CI). A t-test was performed to compare the mean of variables between the two groups. The Kolmogorov-Smirnov test was used for testing the equality of the distribution function of pain scores between the groups. Repeated measures analysis of variance (ANOVA) was performed to test the heart rate and MAP at different points of observation for group and time separately and as the interaction effect of group and time. Missing observations were excluded from the analysis.

## Results

Of the 90 patients recruited for the study, four were excluded, and hence the data of 86 patients were available for the final analysis. The sociodemographic and clinical characteristics of the patients in both groups were comparable, as shown in Table 1.

**Table 1:** Comparison of baseline sociodemographic and clinical characteristics between two groups *Chi-square test. **T-test for the difference in means CI: confidence interval; ASA: American Society of Anesthesiologists

Characteristics	Group V (n=44)	Group D (n=42)	P-value
Gender			0.631*
Male	19	16	
Female	25	26	
Age, years			
Mean (95% CI)	33.9 (30.1–37.7)	37.6 (34.9–40.4)	0.1109**
Weight, Kg			
Mean (95% CI)	57.6 (54.2–60.9)	57.9 (54.6–61.1)	0.91**
ASA status			0.066*
1	36	27	
2	8	15	

Table 2 presents the comparison between the pain scores of patients in the two groups. Pain score was significantly higher among patients in group V as compared to those in group D. The incidence of pain in group V was significantly higher as compared to that in group D. Of note, 76% of patients in the distraction group did not feel any pain while 21% felt mild pain and 2% experienced moderate pain. On the other hand, 4% of patients did not report any pain, 50% reported mild pain, and the rest felt moderate pain in the Valsalva group. No patient reported severe pain in either group. The risk of incidence of pain was four times higher among patients in group V as compared to those in group D [risk ratio (RR) of pain: 4.01, 95% CI: 2.32-6.91, p=0.0001].

**Table 2:** Comparison of pain scores between the two groups *Fisher’s exact test

Score	Group V (n=44)	Group D (n=42)	P-value
0	02	32	0.0001*
1	22	09	0.0001*
2	20	01	0.0001*
3	00	00	0.0001*

Table 3 presents the result of repeated measures ANOVA for the comparison of heart rate between two groups over time. The mean heart rate within each group significantly changed over time in both groups. The heart rate was significantly higher in group V as compared to group D at each observation. There was a significant difference between the heart rate before and after injection. Also, we observed a significant decrease in heart rate over time.

**Table 3:** Analysis of variance for comparison of heart rate over time between the two groups *Adjusted R-squared: 0.1838 d.f.: degree of freedom

Source	Partial sum of square	d.f.	Mean sum of square	F-stat	P-value
Model	9171.12	5	1834.22	12.58	0.0001
Group	5195.75	1	5195.75	35.62	0.0001
Time	2326.03	2	1163.01	7.97	0.0004
Group* time	1606.78	2	803.39	5.51	0.0046

Table 4 presents the result of repeated measures ANOVA for the comparison of MAP between the two groups over time. The MAP within each group significantly changed over time in both groups. The MAP was not significantly different between the groups at each point of observation. There was no significant difference in MAP before and after injection.

**Table 4:** Analysis of variance for comparison of mean arterial pressure over time between the two groups *Adjusted R-squared: 0.2483 d.f.: degree of freedom

Source	Partial sum of square	d.f.	Mean sum of square	F-stat	P-value
Model	4977.52	5	995.50	17.57	0.0001
Group	9.21	1	9.21	0.16	0.6871
Time	4918.11	2	2459.05	43.40	0.0001
Group* time	59.58	2	29.79	0.53	0.5917

## Discussion

As per the main findings of our study, pain scores were significantly higher among the patients in the Valsalva group as compared to those in the lidocaine with distraction group. The incidence of pain also was significantly higher in this group. The risk of incidence of pain was four times higher among patients who performed only VM.

The heart rate was significantly higher among the patients who performed VM both before and after propofol injections. However, there was no significant difference in MAP of patients in the two groups at the baseline, before, and after propofol injection.

These findings are in contrast with the only similar study published so far, that by Kumar et al., who found that VM performed immediately prior to propofol administration attenuates both the severity and incidence of pain without producing any adverse effects [14]. They did not observe any impact of VM on systolic and diastolic blood pressures and heart rates. However, there were some major differences between the methodology of the two studies, which could have influenced the results.

In their study, patients performing VM were compared to those with no intervention. The propofol formulation used by Kumar et al. contained long-chain triglyceride (LCT) whereas we used medium-chain triglyceride (MCT) formulation, which is known to cause lesser pain on injection [14]. No patient in our study experienced severe pain. MCT formulations are associated with less severity of pain on injection as compared to those containing LCT owing to a reduced concentration of free propofol in the aqueous phase [15].

In our study, 20G intravenous cannulae were used whereas they used 18G. The size of the intravenous cannula is critical and can influence the incidence and severity of pain on injection of propofol. We compared VM with the gold standard, which is pretreatment with lidocaine along with distraction by pressing a rubber ball. VM performed by the subjects in their study entailed raising the sphygmomanometer mercury column to 30 mmHg for 20 seconds whereas our patients blew into an aneroid manometer to raise and hold the pressure up to 40 mmHg for 15 seconds.

Pain assessment was done by asking the patients to mark the point on a visual analog scale ruler, and they were asked to fill in a questionnaire to evaluate withdrawal response scores [14]. We used the McCririck pain scale for the assessment of pain, which includes both subjective and objective components and has been widely used for pain assessment associated with propofol injection [11,16].

Limitations of using the visual analog scale for the assessment of propofol injection pain are well known [14]. Visual analog scale scoring may be affected due to propofol sedation, and the numerical interpretation of a single subjective report of transient pain on propofol injection may be misleading. Due to changes in consciousness levels, hand-eye coordination could be impaired in patients, thereby leading to inappropriate responses as well as the loss of data [14]. Another limitation mentioned by the authors includes patients probably having difficulty in finding the point on the visual analog scale line, and cognitive impairments or motor skill issues due to propofol sedation makes this more complicated [14]. None of these issues has been encountered during the use of the McCrirrick pain scale in various published studies. We think that the methodology used in that study greatly influenced the results.

Some studies have shown that VM is effective in alleviating venous cannulation and spinal needle insertion pain [7-10]. It has been suggested that VM attenuates propofol pain by both somatic and distraction mechanisms [14]. Sinoaortic baroreceptor inhibits pain by releasing substance-P into circulation by secreting noradrenaline consequent to compression of thoracic vessels. VM stimulates the vagus nerve, and experimental pain studies have shown that vagus nerve stimulation has an antinociceptive effect in human beings [10]. Pain reduction by VM might be attributed to both pronociceptive and antinociceptive effects triggered by vagus nerve stimulation [17]. It has also been suggested that vagus nerve stimulation might affect peripheral nociceptor functions in humans [18].

During VM, distraction alleviates pain by reducing the level of fear, anxiety, and pain along with increasing the coping capability of patients to procedural pain [19,20]. The activation of the segmental pain inhibitory pathways plays part in pain reduction [21].

The proposed mechanisms of pain produced by propofol injection appear to be quite different and complex as compared to those due to the injection on the body surface as practiced during venous cannulation or for a subarachnoid blockade. This may be a factor responsible for the failure of VM in attenuating this pain.

Various mechanisms have been associated with the pain on injection of propofol, such as endothelial irritation, the difference in osmolarity, non-physiological pH, and the activation of pain mediators [22]. The plasma kallikrein-kinin system is activated by contact with propofol, thereby generating bradykinin. Consequently, the severity of pain aggravates as more free nerve endings outside the endothelial layer of the vessel are now available to come into contact with aqueous-phase propofol. Also, nonselective ligand-gated cation channels like transient receptor potential (TRP), ankyrin1 (TRPA1), and TRP vanilloid 1 (TRPV1) release neuropeptides and act as mediators of propofol-induced pain [23]. The subsequent vascular leakage and dilatation contribute to neurogenic inflammation in the periphery and central sensitization in the spinal dorsal horn.

The results of our study show that neither the somatic nor the distraction mechanism associated with VM was effective in attenuating the propofol injection pain.

The heart rate was significantly higher among the patients in group V as compared to those in group D both before (p=0.001) and after (p=0.001) propofol injections, but there was no significant difference in MAP. It appears that the administration of propofol did not have any significant effect on blood pressure. It also did not mask the changes in heart rate produced by VM. No effect of VM was seen in the study published by Kumar et al. [14]. The hemodynamic variations following VM have been divided into four physiological phases: (I) rise of arterial pressure and a decrease in heart rate, (II) continued strain with a drop in arterial pressure and partial recovery due to reflex tachycardia and vasoconstriction, (III) strain release with a sudden drop in arterial pressure and increased heart rate, and (IV) recovery with arterial pressure overshoot and the resulting bradycardia. It is known that variations in the performance technique of VM greatly influence the pattern of the cardiovascular response to the test [24]. The magnitude of the cardiovascular response correlates with the strain duration, particularly at high levels of strain pressure, and depends on the baseline level of the cardiovascular parameters and their variations. A bradycardic response or opposite effect on blood pressure has also been seen depending on an inspiratory or expiratory VM effort [25].

Our study has several limitations. This was a single-center study involving a limited number of patients. There was no blinding during the interventions. Analgesic effects were not substantiated by biomarker leveling and we did not assess the satisfaction level of patients. Some studies have reported a relationship between the biomarkers calculated from hemogram parameters like neutrophil-lymphocyte ratio and pain perception [26]. Also, variations in the performance technique of VM by the patients may have influenced its success rate.

## Conclusions

Based on our findings, performing VM immediately before the injection failed to attenuate the pain produced by propofol injection as compared to lidocaine pretreatment along with distraction. Neither the somatic nor the distraction mechanism associated with VM was effective in attenuating the pain induced by propofol injection.

## References

[1] Jalota L, Kalira V, George E (2011). Prevention of pain on injection of propofol: systematic review and meta-analysis. BMJ.

[2] Dubey PK, Prasad SS (2003). Pain on injection of propofol: the effect of granisetron pretreatment. Clin J Pain.

[3] McLeod JG, Tuck RR (1987). Disorders of the autonomic nervous system: Part 1. Pathophysiology and clinical features. Ann Neurol.

[4] Randich A, Maixner W (1984). Interaction between cardiovascular and pain regulatory systems. Neurosci Biobehav Rev.

[5] Ghione S (1996). Hypertension-associated hypalgesia. Evidence in experimental animals and humans, pathophysiological mechanisms, and potential clinical consequences. Hypertension.

[6] Carlson KL, Broome M, Vessey JA (2000). Using distraction to reduce reported pain, fear, and behavioral distress in children and adolescents: a multisite study. J Soc Pediatr Nurs.

[7] Agarwal A, Sinha PK, Tandon M, Dhiraaj S, Singh U (2005). Evaluating the efficacy of the valsalva maneuver on venous cannulation pain: a prospective, randomized study. Anesth Analg.

[8] Mohammadi SS, Pajand AG, Shoeibi G (2011). Efficacy of the valsalva maneuver on needle projection pain and hemodynamic responses during spinal puncture. Int J Med Sci.

[9] Kumar S, Gautam SK, Gupta D, Agarwal A, Dhirraj S, Khuba S (2016). The effect of Valsalva maneuver in attenuating skin puncture pain during spinal anesthesia: a randomized controlled trial. Korean J Anesthesiol.

[10] Akdas O, Basaranoglu G, Ozdemir H, Comlekci M, Erkalp K, Saidoglu L (2014). The effects of Valsalva maneuver on venipuncture pain in children: comparison to EMLA(®) (lidocaine-prilocaine cream). Ir J Med Sci.

[11] McCrirrick A, Hunter S (1990). Pain on injection of propofol: the effect of injectate temperature. Anaesthesia.

[12] Smith G, Broek A, Taylor DM, Morgans A, Cameron P (2015). Identification of the optimum vagal manoeuvre technique for maximising vagal tone. Emerg Med J.

[13] Lee JR, Jung CW, Lee YH (2007). Reduction of pain during induction with target-controlled propofol and remifentanil. Br J Anaesth.

[14] Kumar S, Khuba S, Agarwal A, Gautam S, Yadav M, Dixit A (2018). Evaluation of efficacy of Valsalva maneuver for attenuating propofol injection pain: a prospective, randomized, single blind, placebo controlled study. Korean J Anesthesiol.

[15] Hulsman N, Hollmann MW, Preckel B (2018). Newer propofol, ketamine, and etomidate derivatives and delivery systems relevant to anesthesia practice. Best Pract Res Clin Anaesthesiol.

[16] Thukral S, Gupta P, Lakra A, Gupta M (2015). Dexmedetomidine versus ketamine infusion to alleviate propofol injection pain: a prospective randomized and double-blind study. Indian J Anaesth.

[17] Kirchner A, Stefan H, Bastian K, Birklein F (2006). Vagus nerve stimulation suppresses pain but has limited effects on neurogenic inflammation in humans. Eur J Pain.

[18] Kirchner A, Birklein F, Stefan H, Handwerker HO (2000). Left vagus nerve stimulation suppresses experimentally induced pain. Neurology.

[19] Cohen LL (2002). Reducing infant immunization distress through distraction. Health Psychol.

[20] Cohen LL, Bernard RS, Greco LA, McClellan CB (2002). A child-focused intervention for coping with procedural pain: are parent and nurse coaches necessary?. J Pediatr Psychol.

[21] Ong EL, Lim NL, Koay CK (2000). Towards a pain-free venepuncture. Anaesthesia.

[22] Desousa KA (2016). Pain on propofol injection: causes and remedies. Indian J Pharmacol.

[23] Fischer MJ, Leffler A, Niedermirtl F, Kistner K, Eberhardt M, Reeh PW, Nau C (2010). The general anesthetic propofol excites nociceptors by activating TRPV1 and TRPA1 rather than GABAA receptors. J Biol Chem.

[24] Looga R (2005). The Valsalva manoeuvre--cardiovascular effects and performance technique: a critical review. Respir Physiol Neurobiol.

[25] Wong LF, Taylor DM, Bailey M (2004). Vagal response varies with Valsalva maneuver technique: a repeated-measures clinical trial in healthy subjects. Ann Emerg Med.

[26] Tasargol O (2020). A prospective study on the predictability of propofol injection pain. Cureus.

